# Using clinical simulation to evaluate a video telehealth consultation summary application

**DOI:** 10.1038/s41746-026-02506-8

**Published:** 2026-03-11

**Authors:** Teresa O’Brien, Kit Huckvale, Olivia Metcalf, Wendy Chapman, Hasan Ferdous, Rashina Hoda, Peter Poon, Andy Li, Laura Bird, Isabella Hall, Emmy Trinh, Christopher Bain, Sam Georgy, Xiao Chen, Mahima Kalla

**Affiliations:** 1 Digital Health Validitron, Centre for Digital Transformation of Health, Faculty of Medicine, Dentistry and Health Sciences, The University of Melbourne, Carlton, VIC Australia; 2 Faculty of Information Technology, Monash University, Clayton, VIC Australia; 3 Department of Supportive and Palliative Care, Monash Health, Clayton, VIC Australia; 4 Department of Medicine, School of Clinical Sciences, Monash University, Clayton, VIC Australia; 5 Alliance for Digital Health at Monash Faculty of Information Technology, Monash University, Clayton, VIC Australia; 6 Healthdirect Australia, Sydney, NSW Australia; 7 School of Information and Physical Sciences, College of Engineering, Science and Environment, University of Newcastle, Callaghan, NSW Australia

**Keywords:** Health care, Medical research

## Abstract

Patients forget up to 80% of information conveyed during medical consultations. While clinicians may provide hand-written notes to patients during in-person appointments, such opportunities are limited in telehealth. Palliative care patients with complex information needs may benefit from consultation summaries. We developed a consultation summary application (CSA) to generate patient-facing summaries during video telehealth, in a palliative care context. Traditional research methods fall short in early identification and resolution of socio-technical factors, e.g., workflow compatibility, which impact the adoption of digital health innovations. Drawing on the Service Readiness Level Framework, we adopted a phased approach to generating evidence for the CSA. We conducted clinical simulations with seven clinician-simulated patient dyads involving the metastatic lung cancer scenario to examine and address usability and workflow integration issues prior to real-world implementation. Both clinicians and simulated patients perceived the CSA as a valuable tool to support palliative care patients with information recall and self-management. We recommend clinical simulation to de-risk real-world deployment, and optimise the digital health innovations.

## Introduction

Across the world, the prevalence of telehealth services provided through phone or video calls has increased significantly since the COVID-19 pandemic. In 2020 Australia’s Commonwealth Government committed over $100 million towards telehealth optimisation of the development of clinician-facing software applications to facilitate high-quality patient care^1^. Despite growing investment in telehealth globally, an ongoing challenge to optimal patient care is ensuring that clinicians can effectively share medical information during telehealth consultations and that patients can recall it afterwards^2^.

Concerningly, research shows that patients forget or misunderstand 40–80% of the information conveyed during a medical consultation^3–6^. Medical information can be complex, and barriers such as general literacy, health literacy, and digital health literacy further complicate the patients’ accurate recall of medical information. This can negatively impact treatment adherence and consequently, patient outcomes^7^. In an in-person consultation, clinicians can share hand-written notes (e.g., medication instructions and other non-pharmacological lifestyle changes) with patients to help them remember relevant information and take necessary action. In a telehealth consultation, this information-sharing process can be more difficult. For example, variable audio and video quality and lack of opportunity for clinicians to provide hard-copy resources or hand-written notes during the session, may further impact information recall by patients after a telehealth consultation. Preliminary research conducted by Zendel et al. (2021) showed that patients recalled significantly fewer details discussed in telehealth consultations compared to in-person sessions, highlighting a need to bridge the gap in information sharing and retention mechanisms within telehealth consultations^2^.

Certain patient groups, such as those with complex needs or serious illness, may find recalling the content of tele-consultations especially challenging^8^. Our preliminary investigations have highlighted how factors such as fatigue, pain, cognitive effects and memory deficits critically complicate both the effective delivery and experience of telehealth for palliative care patients^9^. In response to this challenge, our consortium of academics, clinicians, industry, and government conducted a series of co-design activities to seek a solution. This culminated in the creation of a consultation summary application (CSA) to supplement Australia’s largest video telehealth platform HealthDirect Australia (HDA). In this project, our academic-clinician-industry-government partnership sought to enhance the Australian video telehealth ecosystem, through responsible design, development, evaluation, and implementation of a fit-for-purpose, patient-centred telehealth application. Further details of the telehealth enhancement application developed in this project are provided in what follows.

HealthDirect Australia (HDA) is a government-funded health information and telehealth service platform used widely across Australian public health services^10^. The HDA video-based telehealth platform is endorsed by the Australian Government Department of Health and Aged Care and the Department of Defence. Our team co-designed a Consultation Summary Application (CSA) to integrate and work well within the HDA platform.

This CSA enables clinicians to type or dictate salient information discussed during a telehealth consultation into an online form, generating a summary that patients can download as a PDF or receive via email. The summary provides a patient-facing record of the consultation, designed to support information recall and self-management after the appointment. It includes pre-populated headings (e.g., background, diagnosis, treatment, medication), which clinicians can tailor if required.

While the clinician is populating information into the CSA form, the patients do not see the summary in real-time until patient view is activated by the clinician. The clinician also has the option to switch off their video camera and microphone while generating a summary. These design choices mitigate the risk of miscommunication from an incomplete summary and affords clinicians some privacy as they complete their task. Clinicians may choose to preview the summary with the patient, discuss and revise its contents as needed, and subsequently provide the final version at the end of the consultation. In accordance with HDA’s data security policy, the CSA does not retain patient or clinical information once the consultation ends.

Another important feature of the CSA is its embedded medical dictionary. To further support patient comprehension, medical jargon is automatically underlined and linked to definitions stored within a medical dictionary embedded within the system’s back-end architecture. These definitions may be viewed by a patient hovering their cursor over the underlined terms in the CSA. Definitions are also included as footnotes in the patient’s PDF summary output. Furthermore, clinicians can edit existing explanations or add new medical definitions for additional terms a patient may require.

Digital innovations show great promise for improving the quality and safety of patient care. However, a large proportion of digital health innovations fail to be adopted into routine clinical practice due to socio-technical challenges such as poor integration with clinical workflows, varying levels of digital literacy among users, and behavioural changes required of patients, clinicians, and administrative staff. A persistent challenge for digital health innovators is that traditional research methods often fall short of identifying and addressing these issues early in the innovation lifecycle, prior to real-world implementation.

Our team sought to anticipate and mitigate potential socio-technical issues which may arise upon implementation of the CSA in the real-world. To this end, we drew on the Service Readiness Level (SRL) Framework which provides a heuristic model for progressively building the evidence base for a digital health innovation from initial problem validation (SRL1) to service implementation at scale (SRL9)^11^. The nine SRLs under this framework include: SRL1–Demand-problem validation and vision; SRL2–Current state (CS) understood/accepted/validated; SRL3–Horizon scanning; SRL4– Future state options co-designed; SRL5–Future state accepted in principle; SR6L–Real world evidence testing; SRL7–Evaluation and evidence gathered; SRL8–Case for scale; and SRL9–Service change implemented^11^.

As part of SRLs1–3, we conducted co-design activities with patients and clinicians, as reported in Kalla et al.^9^ and other internal deliverables. To generate evidence for SRL4, our team undertook think-aloud sessions with clinicians, which will be reported in a forthcoming publication^12^. At SRL5, usability and acceptability testing is required to establish a shared vision of the proposed innovation’s future state. This SRL seeks to ensure that the proposed innovation will be generally compatible with existing work practices and deliver requisite benefits without posing undue burden on clinicians or administrative staff. SRL5 is thus, a critical stage-gate for assessing potential risks and benefits before testing an innovation in the real-world^11^.

We posited that clinical simulation may offer a useful means of testing the CSA at SRL5, before it was sufficiently mature for real-world evaluation. Clinical simulation is an immersive and rigorous method for evaluating emerging digital health innovations in a low-risk environment, prior to real-world testing^13^. It enables researchers to test new innovations within realistic clinical environments to explore issues such as workflow fit, human factors, and trust^14–16^. A key challenge innovators face when trialling new technologies is the personal usage variability inherent within digital health interventions. Thus, a variety of clinical scenarios involving diverse patient and clinician personas may be simulated to better understand user preferences, potential usability challenges, workflow related acceptance and implementation issues, and other human factor considerations^14–16^.

A simulation approach supports psychological safety for participants, and helps improve digital health innovations before they reach patients, especially in sensitive settings such as palliative care^17^. Simulated patient actors, rather than real-life patients, may also be recruited to participate in clinical simulations, to mitigate ethical risks of testing experimental technologies with real-life patients, especially those with limited life expectancy, as may be the case with some palliative care patients.

Our overarching aim was to generate evidence about the usability and acceptability of the CSA under SRL5, and assess its suitability for progression to SRL6–real-world testing. Given the CSA is a clinician-facing interface, our primary objective was to examine and address usability and workflow integration issues from a clinician perspective. Secondly, we explored simulated patients’ perceptions of the potential usefulness of the resulting patient summaries for palliative care patients. This paper serves two purposes: (a) to present findings from the described clinical simulation study; and (b) to share our practical learnings with other researchers interested in using clinical simulation to generate evidence for their digital health innovations.

## Results

### Participants

Seven clinician-simulated patient dyads participated in the study. The mean age for clinician participants was 37 years, with 57% of them identifying as female and remaining 43% as male. The mean age for simulated patients was 50 years, with 29% of them identifying as female and the remaining 71% as male. Further details of clinicians’ and simulated patients’ demographic characteristics, and their pre-simulation questionnaire results are provided in Tables 1 and 2 respectively. An overview of each simulation scenario and dyad is provided in Table 3.

**Table 1:** Clinicians - Participant demographics and responses to pre-simulation questionnaire

Clinician/Gender/ Age	Role title	Current hospital location	How long have you used video telehealth?	Percentage of using video telehealth for consultations in a month?	How often do you use video telehealth in a week? (%)	Application(s) used when conducting video telehealth?	Reflecting on your experience, how would you rate the patient-understanding of the diagnosis or medical conditions after video telehealth consultations?	In your practice, when do patients receive a summary of telehealth consultation?	What are your thoughts on the usefulness of a consultation summary written up in real time?
**C1/F/36**	Medical Oncologist	Metro	2 years	~25%	At least once a week	Healthdirect Australia	There is little room for improvement	Do not receive	Somewhat useful
**C2/M/47**	Palliative Care Specialist	Rural	9 years	~25%	At least once a week	Healthdirect Australia	There is some room for improvement	Within a week to 10 working days, they receive it in post	Somewhat useful
**C3/F/37**	Palliative Care Specialist	Metro	Several years on and off	~25%	At least once a week	Healthdirect Australia	There is some room for improvement	Same time, I share it verbally with them	Very useful
**C4/F/40**	Palliative Care Consultant	Metro	3 years	>75%	At least once a week	Healthdirect Australia	There is some room for improvement	Do not receive	Somewhat useful
**C5/M/32**	Palliative Care Specialist	Rural	3 years	<10%	At least once a week	Healthdirect Australia	There is little room for improvement	Same time, I share it verbally with them	Very useful
**C6/F/27**	Hospital Medical Officer	Metro	1 year	~25%	At least once a week	General video conferencing	There is some room for improvement	Same time, I share it verbally with them, same day, they receive an email/SMS from the clinic/health service	Very useful
**C7/M/38**	Medical Oncologist	Metro	5 years	~25%	Several times a day	Healthdirect Australia, General video conferencing	There is little room for improvement	Same time, I share it verbally with them, same day, they receive an email/SMS from the clinic/health service	Very useful

Notes: General video conferencing = Zoom, Microsoft Teams, WhatsApp, Pexip, Cisco WebEx, Facetime, etc.

**Table 2:** Simulated Patients - Participant demographics and responses to pre-simulation questionnaire

Simulated patient/Gender/Age	Highest education level	How often using video telehealth (%)	Percentage of overall healthcare consultations done as video telehealth	Application(s) used when using video telehealth.	After a telehealth session, do you clearly understand the diagnosis or medical condition discussed?	Do you receive a summary of the consultation after a telehealth session?	If yes, in what form do you receive the summary? (select ALL that apply)	What are your thoughts on the usefulness of a consultation summary written up in real-time?
**P1/M/70**	Bachelor’s degree or equivalent	Once a fortnight	~50%	HealthDirect Australia	Yes	Occasionally, depending on the circumstances, and if so, I received it within 24 hours	SMS	Very useful
**P2/F/58**	Bachelor’s degree or equivalent	Once or twice a year	~25%	HealthDirect Australia, General video conferencing	Partially, I have some understanding but would like further clarification	No, rarely or never and if so, I received it within a week	Email, via my GP	Very useful
**P3/M/42**	Advance Diploma in the Arts	Once every six months	~50%	HealthDirect Australia	Yes	Occasionally, depending on the circumstances	Email, SMS	Somewhat useful
**P4/M/76**	Early school leaver	Only once	<10%	Others	Yes	No, rarely or never	Verbal	Very useful
**P5/M/21**	High school diploma or equivalent	Never	<10%	Not applicable	Not applicable	Occasionally, depending on the circumstances	Email	Very useful
**P6/F/30**	Master’s degree or equivalent	Only ever used it once	<10%	HealthDirect Australia	Not applicable	Occasionally, depending on the circumstances; and if so, I received it immediately	Email	Somewhat useful
**P7/M/50**	Bachelor’s degree or equivalent	Once per year	<10%	Others	Yes	No, rarely or never	Email	Very useful

Notes: Simulated patients responded based on their own prior experiences (not as their assigned personas). GP = General Practitioner. General video conferencing = Zoom, Microsoft Teams, WhatsApp, Pexip, Cisco WebEx, Facetime, etc.

**Table 3:** Clinical simulation dyads and scenarios

Simulation Session#	Clinician-simulated patient IDs	Key patient persona details and consultation purpose	Initial or follow-up visit
1	C1-P1	70 years old; male; no longer working; Manage side effects of constipation from use of morphine to manage pain.	Initial
2	C2-P2	50 years old; female; software engineer, feeling like pain and prognosis has affected relationship with work (falling behind and letting team down); needs help managing pain and poor sleep.	Initial
3	C3-P3	40 years old; male; pianist by profession, lives with 75 years old mother; wishes to explore available services and support options for home care.	Initial
4	C4-P4	70 years old; male; live alone; struggle with insomnia and low appetite; needs help managing appetite	Follow-up
5	C5-P5	35 years old; male; needs help with managing poor sleep, likely due to anxiety	Follow-up
6	C6-P6	40 years old; female; taking morphine to manage pain, which is causing side effects such as constipation; needs help managing medication side effects	Follow-up
7	C7-P7	50 years old; male; taking morphine to manage pain, which is causing side effects such as constipation; needs help managing medication side effects	Follow-up

Note: All personas centre around advanced-stage lung cancer where chemotherapy and radiotherapy treatments have been completed, but the disease has progressed to a point where further curative treatment is no longer an option.

### Technology acceptance

The acceptance data from clinicians’ and simulated patients’ post-simulation questionnaires are provided in Tables 4 and 5 respectively. Average acceptance score was 74% for clinicians, and 94% for simulated patients. Clinicians rated the CSA as intuitive and accessible (ease of use: 82%) and expressed willingness to adopt it when it aligns with clinical needs (intention to use: 72%), despite noting concerns about increased cognitive load and workflow disruption. Simulated patients rated usability and usefulness highly (96%), with strong scores for terminology clarity, presentation, and trustworthiness.

**Table 4:** Clinicians’ acceptance of the CSA

Construct (number of questions asked)	Mean Score	Mean score in %
**Performance expectancy** (3)	3.0	60
**Effort expectancy** (4)	4.1	82
**Attitude** (4)	3.6	72
**Facilitating conditions** (3)	3.9	78
**Self-efficacy** (4)	3.8	76
**Anxiety** (4)	4.0	80
**Behavioural intention** (3)	3.6	72
**Average**	**3.7**	**74**

**Table 5:** Simulated patients’ acceptance of the summary output

Aspect (number of questions asked)	Mean Score	Mean Score in %
**Summary document** (3)	4.9	98
**Summary feature** (2)	4.8	96
**Terminology explanation** (2)	4.6	92
**Terminology presentation** (3)	4.7	94
**Generated summary** (2)	4.6	92
**Overall** (3)	4.5	90
**Average**	**4.7**	**94**

Based on the simulations and debrief sessions, opportunities for improving the usability of the CSA were identified. In summary, we implemented the following improvements: (i) voice dictation was not found helpful by clinicians, therefore we introduced a toggle button to make it optional; (ii) clinicians requested support for rich text formatting, including the ability to add tables for listing medications and their uses which we implemented; (iii) to give clinicians more control over ensuring patients receive the summary, we added a feature that allows them to send it via email, rather than relying solely on patients to save or print the summaries; (iv) clinicians experienced frustration at not being able to adjust the video size of the patient, so we enabled drag-and-resize functionality; (v) clinicians and simulated patients found some word definitions (e.g., “chemotherapy”) too lengthy, thus we introduced a character limit to improve readability and reduce clinicians’ time to proofread. A table summarising the changes made to the CSA user interface, in response to the study findings is also provided in Supplementary Materials no.5. The following section provides qualitative insights into clinicians’ and simulated patients’ perspectives on the CSA and its summary outputs.

### Clinician perspectives on perceived value and use cases

During the simulated consultations, clinicians used the CSA to document information such as symptoms (*n* = 5), create symptom management plans (*n* = 6), share resources such as web links (*n* = 1), and describe medical terms that patients might struggle to understand (*n* = 6). Most clinicians (*n* = 6) previewed their summaries with patients to explain their notes and refine them if needed. This also afforded patients the opportunity to ask follow-up questions before downloading the final summaries. Generally, all clinicians (*n* = 7) acknowledged the potential value of consultation summaries for patients.

Most clinicians felt that not all patient consultations would require a summary output, especially short consultations with straightforward takeaways. Some believed that routine use of CSA could ensure more consistent care, especially for palliative care patients with complex needs. The CSA enabled clinicians to share notes and web links in a retrievable and organised way, prompting some to consider sharing online resources they might not have otherwise provided.

Clinicians considered the CSA as a more formal method of sharing information with patients, compared to chat, email, or hand-written notes. Additionally, they noted that if the summary document could be shared with other clinicians, it could help them understand what had been previously communicated to the patient. Generally, clinicians did not see additional risks associated with the typed summaries compared to providing hand-written notes during in-person consultations.

### Clinician perspectives on workflow integration

While the clinicians had been provided a mock EMR to replicate their routine clinical practice, they did not use the EMR during the clinical simulation. Thus, the flow of information between the EMR and CSA could not be investigated further. Nevertheless, during the debrief interviews, clinicians noted that integrating the CSA with EMRs could be beneficial in the long run.

Prior to preparing their consultation summaries, most clinicians chose to let patients know that they would be switching off their videos and muting their microphones as they developed the summaries. This helped avoid any awkwardness and allowed the clinicians some privacy as they prepared the summaries for patients. One clinician (C4) chose not to use the CSA during the simulated consultation at all, feeling it was awkward to switch off audio and video, and type while the patient remained on the call. During the debrief interview, they explained that they typically write notes after a consultation, making in-consultation use of the CSA incompatible with their usual practice.

Clinicians agreed that integrating a new application into routine workflows would require practice and greater cognitive load, especially during the initial adjustment period. For example, when generating the summaries, clinicians had to ensure that their notes were clear for a public audience, unlike EMR notes where medical jargon is acceptable. This tidy-up and proof-read process typically took clinicians an average of 2–7 min during the simulations. Subsequently, previewing the summary with patients also took additional time to allow patients to read, digest, and ask follow-up questions prior to downloading their summaries. All these steps are not part of the current workflows and can be expected to add to consultation length. Nevertheless, clinicians felt that sharing summaries during consultations, rather than days or weeks later, would help avoid delays or omissions, and support better patient care—a trade-off that may be worth making.

### Simulated patients’ perspectives on perceived usefulness of summaries

All simulated patients felt that the summaries would be helpful for real-life patients, especially those with chronic illnesses. One older simulated patient (P4; aged 76) commented that the summary would help him personally, with information retention, reduce reliance on memory, and support timely follow-up action for self-management. Simulated patients also viewed the summary as a helpful tool for patients to communicate relevant information to their family members or other informal caregivers.

Simulated patients generally agreed that the medical definitions would be helpful. They perceived the bespoke definitions added by clinicians as more relatable and trustworthy, compared to those from the embedded medical dictionary—a few of these default definitions were considered long and daunting.

Simulated patients also noted that the usefulness of summaries may vary across patients. For example, they felt that newly diagnosed patients may have greater information needs and thus benefit more from receiving the summaries.

One simulated patient commented on the implications of repeat CSA use during a real-life patient’s care journey. They noted that it would be the patient’s responsibility to destroy or archive past summaries to avoid confusion, particularly when treatment options or medication dosages had been adjusted by the clinician.

### Simulated patients’ perspectives on experience of receiving and accessing summaries

Due to privacy considerations, the CSA is set up so that summaries are lost once the consultation ends, requiring patients to download them during the video call. Five of the seven simulated patients downloaded their summaries before their simulated consultations ended. One simulated patient (P2) was not prompted to do so by their clinician. The video call ended before the simulated patient had downloaded their summary—highlighting a potential source of frustration for real-life patients. The simulated patient (P4), whose clinician (C4) did not use the CSA during the simulated consultation, did not receive a summary, and thus could not comment on the experience of accessing one.

Simulated patients whose clinicians previewed the summaries and provided them an opportunity to digest the information and ask questions during the consultation spoke positively about this experience. They found it value-adding, felt more included in their care, and believed it could strengthen the therapeutic alliance between clinicians and patients.

Simulated patients noted that they needed to wait while clinicians prepared the summaries, with one (P5) describing feeling ‘awkward’ or ‘bored’ while waiting without clear guidance on whether it was acceptable to use their phone, for instance, to pass the time. From a real-life palliative care perspective, some simulated patients expressed concern that waiting could be somewhat uncomfortable for palliative care patients experiencing tiredness, pain, or other discomfort.

## Discussion

In this study, we aimed to explore the usability and acceptability of a Consultation Summary Application (CSA) to support palliative care patients with post-consultation information recall and self-management. Given the ethical ramifications of testing an emerging technology with a vulnerable patient group with limited life expectancy, we used a clinical simulation approach. Our simulation approach involved mock clinical encounters between real-life clinicians and simulated patients based on a pre-determined clinical scenario and patient personas. This approach helped us test and refine the CSA, and assess its suitability for progression to real-world evaluation, in line with the phased evidence generation approach proposed by Hughes et al.’s (2021) Service Readiness Level Framework^11^.

Quantitative and qualitative data showed that clinicians and simulated patients generally perceived potential benefits of a patient-facing summary, while identifying usability issues that informed further optimisation of the application. (A complete list of refinements made to the CSA in response to participant feedback is provided in Supplementary Materials no.5).

In our estimation, design of credible clinical simulations requires requisite attention to socio-technical complexities inherent within divergent and often fragmented clinical workflows. Practitioners interested in the simulation method thus need to consider a myriad of pragmatic and logistical factors to design and execute studies that will yield actionable insights, capable of advancing the innovation process. Herein, we provide a deeper exploration of our experience of conducting clinical simulation. We offer recommendations for other digital health innovators who may wish to trial or optimise this research method in their practice. The following pragmatic insights are salient methodological considerations intended to enhance the rigour and relevance of clinical simulation research in digital health innovation.

Our study aligns with the methodological guidance provided by Jensen, Kushniruk, and Nohr (2015)^18^, particularly regarding the importance of rehearsals for successful clinical simulation. We conducted four rounds of rehearsal to refine our protocol. The first rehearsal focused on testing and optimising the technical setup for the simulations. This included configuring the simulation environment to nest HealthDirect Australia video telehealth calls within Zoom, allowing researchers to observe and manage sessions while also enabling clinicians and simulated patients to interact within a realistic telehealth setting. The second rehearsal focused on developing participant briefing instructions, which were captured in end-to-end run sheets to guide researchers in supporting clinicians and simulated patients during the sessions. Copies of these run sheets are provided in Supplementary Materials no. 6. This rehearsal involved a palliative care specialist (co-author PP) as the clinician and a researcher (independent from the project team) as the simulated patient. The third rehearsal served as a pilot test of the technical setup and the participant briefing materials prepared in the previous rehearsals, with authors OM and TOB playing the respective roles of clinician and simulated patient. The fourth and final rehearsal was a complete high-fidelity run-through with a clinician-researcher (independent from the project team) as the clinician and author TOB as the simulated patient.

Across these rehearsals, the need for dedicated technical support became evident. Participants occasionally encountered device configuration and browser compatibility issues that researchers could not always resolve. Having a dedicated technical support person (co-author AL) on standby ensured that technical issues could be promptly addressed, allowing simulations to progress efficiently.

Assigning a designated researcher ‘buddy’ to ‘handhold’ each clinician and simulated patient proved essential. Given the number of steps involved in the simulations, both for technical and research purposes, the experience may potentially feel stressful for some participants. Thus, having a researcher guide each participant from onboarding to the de-brief interview fostered a sense of comfort and support for the participants.

Pacing and iterative refinements have enabled the software development team to troubleshoot any technical glitches encountered in the CSA during the simulation sessions. A few days were allowed between consecutive simulation sessions. These breaks enabled the research team to debrief and refine the protocol for subsequent sessions, such as making minor adjustments to participant briefing instructions to improve their study experience. This cadence also enabled the software development team to troubleshoot any technical glitches encountered in the CSA during the simulation sessions.

Our initial goal was to run simulations using both first-visit and follow-up appointment scenarios. However, we found that first-visit simulations required clinicians to spend considerable time taking patient histories, leaving little time to use and test the CSA. We therefore transitioned to follow-up visit scenarios only. To make this effective, researchers pre-entered information into the mock EMR to avoid redundant questioning, and simulated patients were thoroughly briefed on these notes to ensure consistent responses during clinician questioning in the mock consultations.

We prioritised diversity by involving clinicians from varied geographic (rural and metropolitan) and professional backgrounds (oncologist, palliative care specialist, junior doctor) with differing levels of video-telehealth experience. We also included simulated patients of varied ages, genders, and culturally and linguistically diverse backgrounds (Australian, Persian, Zimbabwean, Malaysian), which augmented the simulation.

Despite the strengths of this study, several limitations and future directions should be considered. Clinicians interacted with the CSA for the first time during the clinical simulation. Prior to commencing the mock consultations with simulated patients, clinicians were asked to watch a demonstration video and practise using the CSA. This initial trial surfaced usability challenges typical of first-time use due to a lack of familiarity with the user interface. However, some of the issues identified during a single simulation session may not persist with repeated exposure to the technology. To confirm this, future simulation studies may incorporate repeated-use scenarios. Findings from the simulation can inform training needs, in which clinical simulation itself also offers an effective educational approach for preparing clinicians before real world implementation.

The primary aim of our study was to assess CSA usability from a clinician perspective, as this is a clinician-facing tool. We therefore recruited simulated patients rather than real-life patients. We recognise, however, that simulated patients are limited in the depth of feedback they can provide during debriefing. The tacit knowledge that a real-life patient possesses due to their lived experience, but which a simulated patient may lack, has implications for simulation fidelity. As such, simulated patients may require further pre-simulation training to equip them with the necessary medical knowledge that a real-life patient may otherwise possess, such as medication dosing and frequency for their ongoing medication. Nevertheless, given our primary research question, simulated patient perspectives were sufficient for our purposes.

While we sought to achieve diversity among clinician and patient dyads, future simulations could further expand inclusivity by incorporating triads, such as patient-clinician-interpreter, or patient-clinician-carer. Although data saturation was reached for usability issues in our study, broader representation in patient and clinician personas may yield additional perspectives and enhance the generalisability of findings for similar future technologies and simulation studies. Additionally, no gender diverse individuals volunteered to participate, and sexual orientation data were not collected. Exploration of other issues and their design implications, such as contextual privacy in video telehealth, which may disproportionately impact certain patient communities (e.g. LGBTIQ+ patients) could be the subject of a future study.

This study demonstrates the potential value of using clinical simulation to evaluate the usability and acceptability of a video-telehealth consultation summary application prior to conducting real-world clinical testing. Our findings indicate that both clinicians and simulated patients perceived the CSA as a useful tool to support information recall and self-management, particularly in the palliative care context where patients often face complex information needs. The simulation method also provided a psychologically safe, low-risk environment to engage simulated patients as end-users while maintaining ethical sensitivity, especially when working with potentially vulnerable patient populations. We intentionally sought socio-demographic diversity in our participant sample to enhance the inclusivity of our clinical simulation design.

Insights gathered from each session directly informed iterative improvements, enhancing the CSA’s relevance and usability. The clinical simulation approach served two key purposes: (a) it offered reassurance to the team that the CSA, once refined based on the simulation findings, would justify progression to real-world testing; and (b) it enabled early identification and mitigation of potential points of frustration or failure that could discourage clinicians from using the CSA with real-life patients during a future clinical study, potentially leading to participant attrition and disuse of the application.

Our experience highlights the utility of clinical simulation for advancing digital health innovations through structured, phased evidence generation, as articulated by the Service Readiness Level Framework. Clinical simulation may warrant consideration by other digital health innovators as a potentially helpful approach to de-risk clinical studies and real-world implementation, support design refinements of emerging technologies, and contribute to phased evidence generation for digital health innovation pipelines.

## Methods

### Simulation environment

**Fig. 1:**
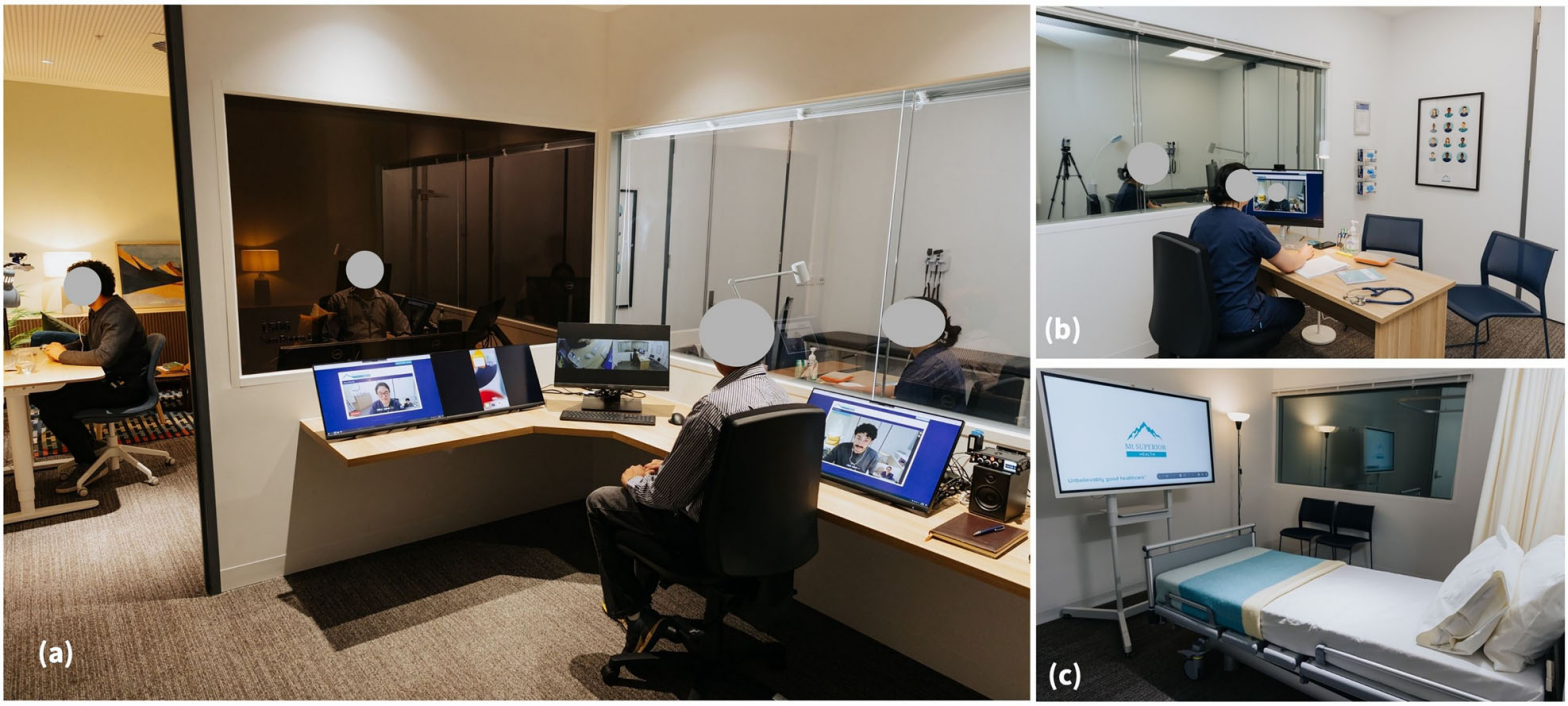
The clinical simulation environment at the Validitron, Centre for Digital Transformation of Health. **a** left side – simulated patient situated in the simulated patient home space having a telehealth consultation with clinician; right side – human-computer interaction researcher situated in the control room, observing the proceedings both via one-way glass and screen mirroring; **b** clinician situated in the simulated primary care room conducting a telehealth consultation with patient next door; **c** example of another tertiary care simulated space in the laboratory (not utilised in this particular simulation study).

The clinical simulation study was conducted at the Validitron, Centre for Digital Transformation of Health Simulation Laboratory. The Validitron laboratory contains a physical suite of themed home, primary and secondary care rooms where clinicians and patients can co-design and test tools in a realistic, but controlled environment (see Fig. 1). The laboratory is optimised for clinical simulation studies and human factors research. Each simulation room has a covert observation area behind one-way glass with digital video, audio, and screen recording capabilities.

Complementing the laboratory, the Validitron Sandbox provides a digital replica of a health information ecosystem, built on established and emerging data standards such as Fast Healthcare Interoperability Resources (FHIR®). The Sandbox incorporates a range of production, Open Source and white label healthcare information systems such as instances of primary and secondary care Electronic Medical Records (EMRs), telehealth platforms, and synthetic data creation tools. These components can be combined like LEGO® bricks to rapidly build virtual infrastructure within which new tools can be tested for workflow and technical compatibility. This combination of the physical space and virtual Sandbox provides an immersive, end-to-end environment to accelerate user testing and optimise the real-world implementation readiness of digital health innovations.

An example clinical simulation setup for illustrative purposes only, is provided in Fig. 1. For the current study, clinicians participated remotely from their home environments using personal devices. Simulated patients participated in-person from our laboratory space. The virtual Sandbox environment for this study comprised the HDA telehealth platform and an EMR form, to replicate the clinical workflow of palliative care video telehealth consultations.

### Simulation scenario and personas

The research team developed an overarching clinical scenario involving the diagnosis of metastatic stage-4 lung cancer, in which curative treatment was no longer an option. The scenario addressed common concerns in palliative care, including management of morphine-related side effects (e.g., constipation), poor sleep, loneliness, anxiety, exertional breathlessness, and interest in community support resources.

Different patient personas were created to reflect a variety of ethnicities, jobs, hobbies, and living situations. Further details of sample patient personas used for the simulations are provided in Supplementary Materials no.1. The scenario and personas were reviewed by a palliative care specialist (author PP) for clinical validity and relevance to typical palliative care presentations. Clinical simulations encompassed both newly referred and follow-up patient consultations. For new patient personas, an accompanying mock referral letter was also provided to clinicians, a sample of which is available in Supplementary Materials no.2.

### Participant criteria and recruitment

Clinicians were eligible to participate if they were based in Victoria (Australia) and had prior experience of conducting video-based telehealth consultations with palliative care patients. Clinician recruitment was conducted through author PP’s professional networks, snowball sampling, and a third-party participant recruitment agency (*Askable*).

To avoid ethical ramifications of recruiting a vulnerable patient community with limited life expectancy to test a developing technology, our team decided to recruit professional actors with experience of playing patients for medical education and research purposes. Given the CSA is a clinician-interfacing technology and our primary research aim was to test usability issues from a clinician perspective, our team deemed it sufficient to recruit trained simulated patients for this pre-clinical evidence generation.

Simulated patients were recruited through The University of Melbourne Simulated Patient Program. Simulated patients were eligible to participate if they were aged 18 years or older. Simulated patients expressing interest in participating in the study were selected based on the persona requirements (e.g. age, gender, cultural heritage etc.). A total of seven clinician-simulated patient dyads were recruited; participant information is provided in the results section.

### Simulation procedure and data collection

**Fig. 2:**
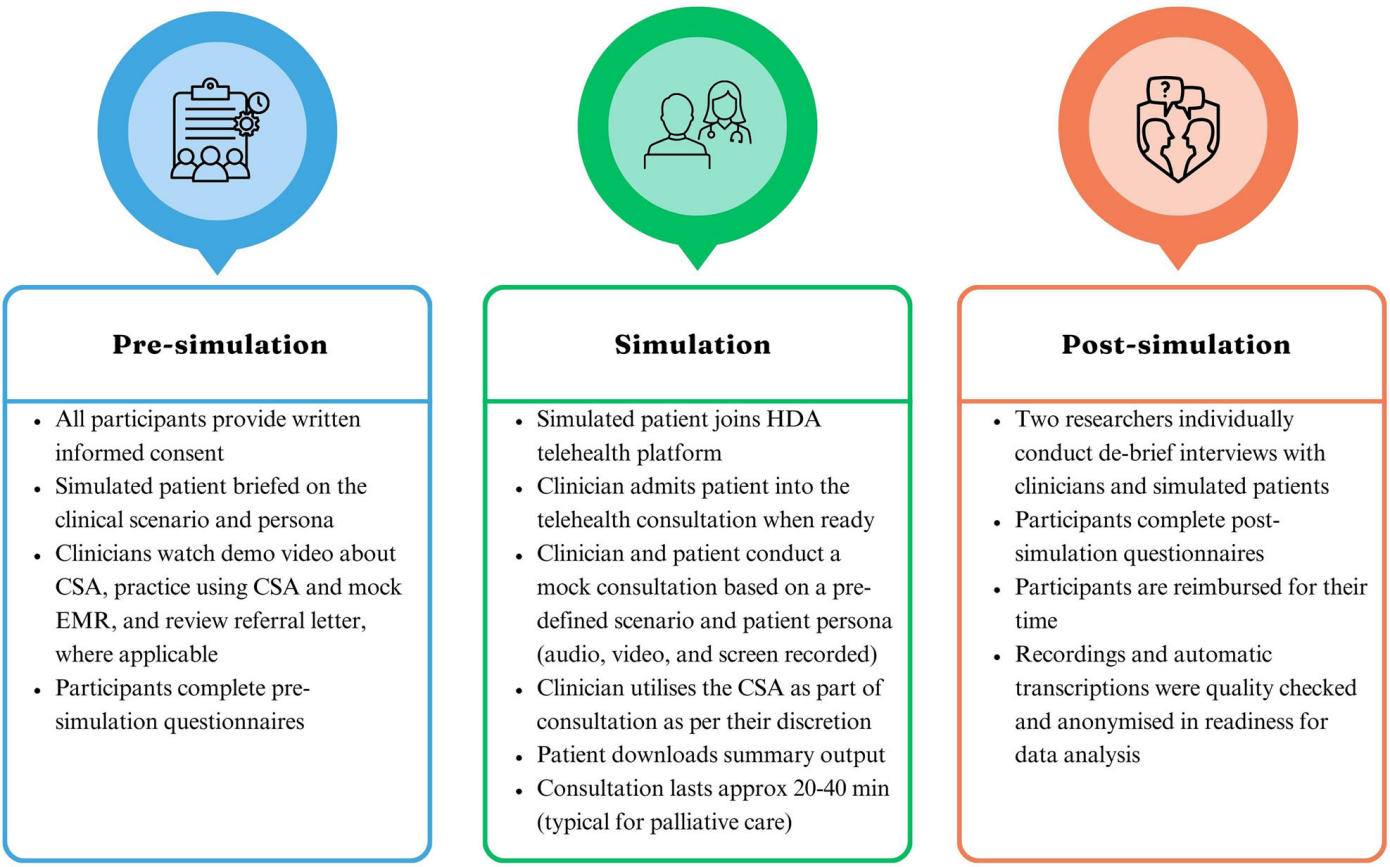
An overview of key activities conducted within each phase. Figure created by the authors using Canva software.

The clinical simulation study was conducted in three phases: pre-simulation, simulation, and post-simulation, with each simulation session typically lasting approximately 90 min. An overview of key activities conducted within each phase is provided in Fig. 2. Prior to the simulated consultation, clinicians completed a consent form, a questionnaire about their demographic characteristics, and views and experience of video telehealth. Simulated patients completed a consent form, a questionnaire about their demographic characteristics, and past experience of using telehealth for their personal healthcare (**not** their assigned patient personas). After the simulation, the clinicians and simulated patients were interviewed separately by two researchers and asked to complete post-simulation questionnaires. Clinician questionnaires were based on an abridged version of the Unified Theory of Acceptance and Use of Technology (UTAUT) and the Technology Acceptance Model (TAM), assessing constructs such as performance expectancy, effort expectancy, social influence, facilitating conditions and technology adoption^19,20^. Simulated patients completed a bespoke questionnaire about their experience of the CSA-generated summary, covering aspects such as readability, accuracy, level of detail, overall satisfaction, and perceived usefulness for real-life patients. All questionnaires and semi-structured interview guides are provided in Supplementary Materials no.3 and no.4 respectively.

**Fig. 3:**
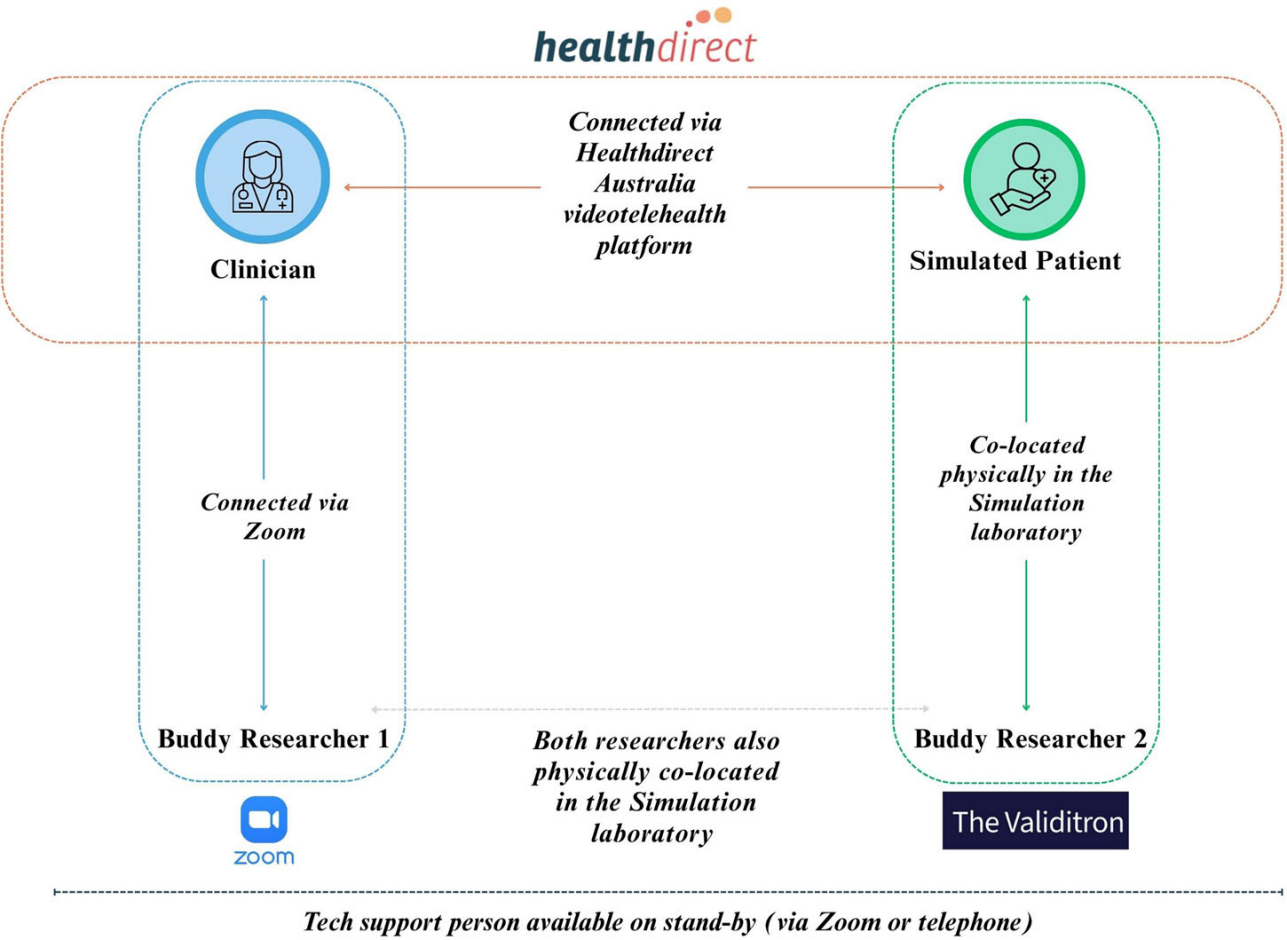
Connections between clinician, simulated patients, and researchers. Figure created by the authors using Canva software.

One researcher guided the clinician via Zoom, while another assisted the simulated patient in-person at our simulation laboratory. A dedicated technical support person was present on stand-by throughout the simulation to help troubleshoot any emerging technical issues (see Fig. 3).

### Data analysis

Two Authors TOB and MK, reviewed one dyad of a simulated consultation along with the debrief interviews, watching the recordings and analysing the data for the dyad together. They reflected on insights related to usability issues, workflow compatibility, summary readability, and medical terminology explanations. The emergent insights with representative participant quotes were captured in an internal memorandum. Subsequently, Author TOB reviewed the other six dyads’ data replicating the process as described. Finally, author TOB synthesised the emergent insights, distiling the results into three categories: (1) clinicians’ perceived benefits; (2) clinicians’ perceived challenges; and (3) simulated patients’ perceptions about the CSA summary outputs.

### Ethical approval and participant compensation

Ethical approval was obtained from the University of Melbourne Human Research Ethics Committee (Project number: 28506). Upon completing the study, each clinician received a financial compensation of $150, while the simulated patients were remunerated according to their regular professional hourly rate of $80 per hour.

## Supplementary information

COMMSMED-25-1668-T-s02

## Data Availability

The datasets generated during and analysed during the study can be requested via the corresponding author.
