# Supplementary material for: Using clinical simulation to evaluate a video telehealth consultation summary application
Source: NPJ Digit Med. 2026 Mar 11;9:415. doi: 10.1038/s41746-026-02506-8 (PMC13230944; doi:10.1038/s41746-026-02506-8)
Supplement: Supplementary file 1 — COMMSMED-25-1668-T-s02 [file 41746_2026_2506_MOESM1_ESM.pdf]

Supplementary Materials

No. 1. Patient Personas used for Clinical Simulations

If the clinician is a **medical oncologist,** see **A.** below**.**

A
Pain point:
Morphine use – side effects of constipation.

Background:
I am diagnosed with advanced-stage lung cancer with a history of heavy smoking. I have had previous treatment (chemotherapy and radiation) with the primary location in the right upper lung and metastasis to the right sided ribs. I have just completed further radiotherapy for the painful ribs and am now present to the doctor as pain remains poorly controlled.

To manage my severe rib pain, my morphine dose has recently been increased to 30 mg twice a day, and I take additional liquid morphine as needed, up to 3 – 4 times a day (10mg each time). My pain score typically reduces from 7 to 2 after taking it. However, I'm conscious of the risk of becoming too dependent on morphine, and the constipation and nausea I sometimes experience make me hesitant to take it.

If the clinician is a **palliative care specialist,** see **B.** below**.**

B
Pain point:
Poor sleep (lonely/anxious), some mild shortness of breath on exertion, interested in resources for community support.

Background:
I am diagnosed with advanced-stage lung cancer with a history of heavy smoking. I have been undergoing treatment (chemotherapy and radiation) but the disease has progressed to a point where further curative treatment is no longer an option.

I currently don't have any pain and am on some regular Panadol (no opioids required). I feel lonely and am sleeping poorly because I wake up often and my mind races, it's frustrating. I do have some shortness of breath when trying to do some gardening but otherwise I am coping to care for myself at the moment. I don't have any community services engaged, and my GP does not do home visits.

No. 2. Supplementary Figure 1: Sample Mock Referral Letter to Accompany New Patient Personas

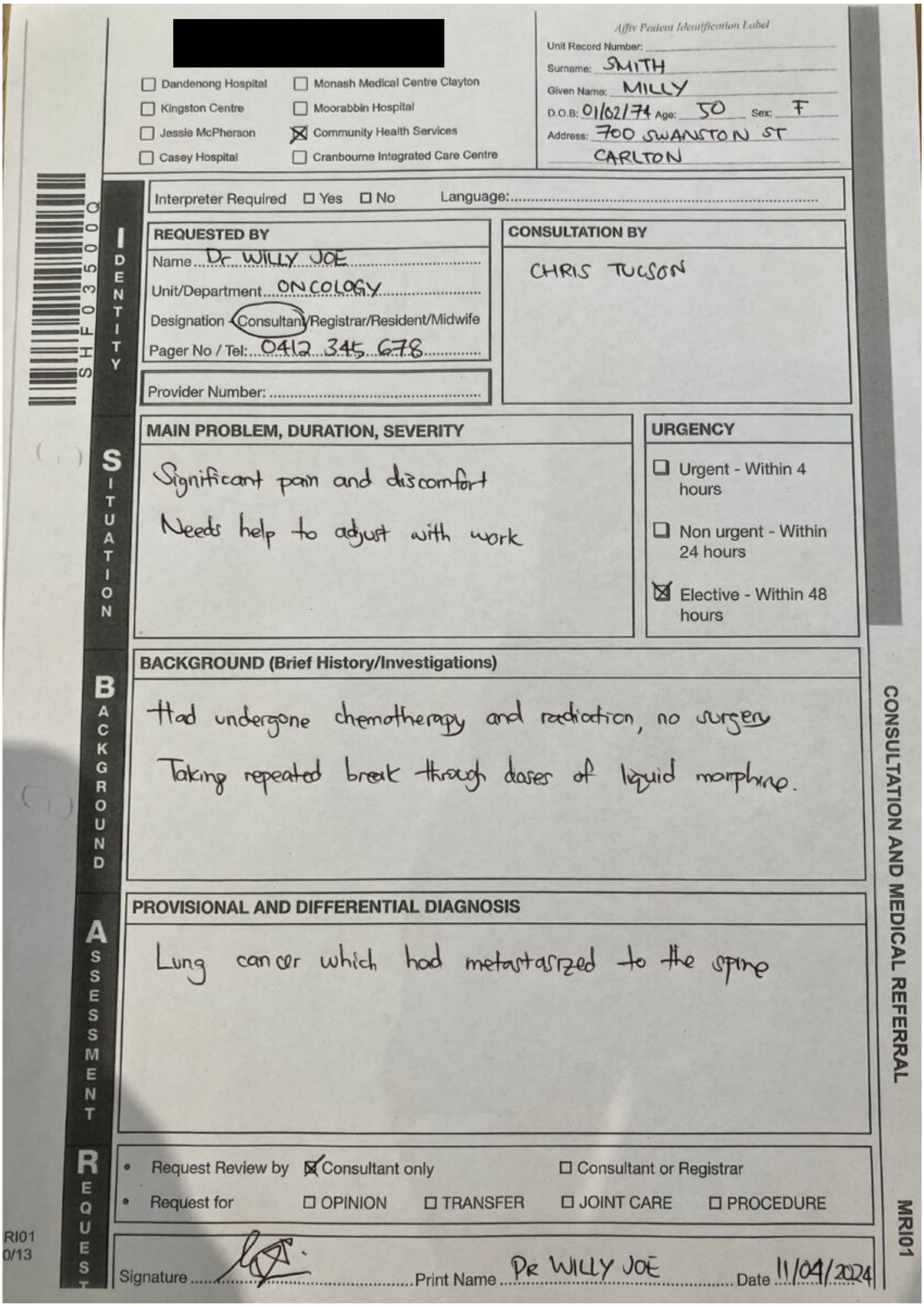

[redacted]

- ☐ Dandenong Hospital
- ☐ Kingston Centre
- ☐ Jessie McPherson
- ☐ Casey Hospital
- ☐ Monash Medical Centre Clayton
- ☐ Moorabbin Hospital
- ☒ Community Health Services
- ☐ Cranbourne Integrated Care Centre

Affix Patient Identification Label

Unit Record Number:

Surname: SMITH

Given Name: MILLY

D.O.B: 01/02/74 Age: 50 Sex: F

Address: 700 SWANSTON ST CARLTON

SHF035000

Interpreter Required ☐ Yes ☐ No Language:

IDENTITY

REQUESTED BY

Name: Dr WILLY JOE

Unit/Department: ONCOLOGY

Designation: (Consultant)/Registrar/Resident/Midwife

Pager No / Tel: 0412 345 678

Provider Number:

CONSULTATION BY

CHRIS TUCSON

SITUATION

MAIN PROBLEM, DURATION, SEVERITY

Significant pain and discomfort
Needs help to adjust with work

URGENCY

- ☐ Urgent - Within 4 hours
- ☐ Non urgent - Within 24 hours
- ☒ Elective - Within 48 hours

BACKGROUND

BACKGROUND (Brief History/Investigations)

Had undergone chemotherapy and radiation, no surgery
Taking repeated break through doses of liquid morphine.

ASSESSMENT

PROVISIONAL AND DIFFERENTIAL DIAGNOSIS

Lung cancer which had metastasized to the spine

REQUEST

- Request Review by ☒ Consultant only ☐ Consultant or Registrar
- Request for ☐ OPINION ☐ TRANSFER ☐ JOINT CARE ☐ PROCEDURE

Signature [signature] Print Name Dr WILLY JOE Date 11/04/2024

RI01 0/13

CONSULTATION AND MEDICAL REFERRAL

MRI01

Supplementary Figure 1. Sample mock referral letter to accompany new patient personas.

No. 3. Questionnaires

<u>Pre-simulation Questionnaire for Clinicians</u>

Demographic

1. What was your age on your last birthday?
2. What was your sex recorded at birth? (select one)
    a. Male
    b. Female
    c. Prefer not to say
    d. Another term (please specify): ________
3. Briefly, what is your professional qualification and current role?
4. How many years of Palliative Care experience do you have?

Telehealth Experience

5. In a usual month, what percentage of your overall healthcare consultations are done as telehealth video consultations?
    a. Less than 10%
    b. Around 25%
    c. Around 50%
    d. Around 75%
    e. More than 75%
    f. 100%
6. How often do you, as a healthcare provider, utilise telehealth (in the form of video calls)?
    a. Several times a day
    b. At least once a day
    c. At least once a week
    d. At least once a month
    e. Once or twice in a year
    f. Never
7. What applications do you use for your telehealth consultation? (select all that apply)
    a. Healthdirect Video Call
    b. General video conferencing (e.g. Zoom, Microsoft Teams, Whatsapp, Pexip, Cisco WebEx, Facetime, etc.)
    c. Others (please specify): ____________
8. In your experience, how would you rate the understanding of the diagnosis or medical conditions your patients have after the telehealth video consultations?
    a. They have perfect understanding
    b. There is a little room for improvement
    c. There is some room for improvement
    d. There is significant room for improvement
9. In your experience, how long does it take for the patients to receive a summary of their telehealth consultation with you? (select ALL that apply)
    a. Patients do not receive a summary
    b. Same time, I share it verbally with them
    c. Same day, they receive an email/SMS from the clinic/health service
    d. Within a week to 10 working days, they receive it in post.
    e. Other (please specify):____________
10. Imagine your telehealth patients and carers can receive a relevant summary of the consultation written up in real-time by the end of your video call with them. How useful do you think it will be for them?
    a. Not at all useful
    b. Not really useful

c. Somewhat useful
d. Very useful

Pre-simulation Questionnaire for Simulated Patients
Demographic

1. What was your age on your last birthday?
2. What was your sex recorded at birth? (select one)
   a. Male
   b. Female
   c. Prefer not to say
   d. Another term (please specify): ________
3. What is your highest level of education?
   a. High school diploma or equivalent
   b. Bachelor's degree
   c. Master's degree
   d. Doctorate degree
   e. Other (please specify) ________

Telehealth Experience

4. In a usual month, what percentage of your overall healthcare consultations are done as telehealth video consultations?
   a. Less than 10%
   b. Around 25%
   c. Around 50%
   d. Around 75%
   e. More than 75%
   f. 100%
5. How often do you use telehealth (via video calls)?
   a. Several times a day
   b. At least once a day
   c. At least once a week
   d. At least once a month
   e. Once or twice in a year
   f. Never
6. After a telehealth session, do you feel that you clearly understand the diagnosis or medical condition discussed?
   a. Yes, I clearly understand the diagnosis or medical condition discussed.
   b. I understand partially but would like further clarification.
   c. No, I do not understand clearly and need additional explanation.
   d. Not applicable; a diagnosis or medical condition was not discussed during the session.
7. Do you receive a summary of the consultation after a Telehealth session?
   a. Yes, always
   b. Occasionally, depending on the circumstances
   c. No, rarely or never

7a. If so, when will you receive the summary?
   a. Immediately after the Telehealth session
   b. Within 24 hours after the Telehealth session
   c. Within a week after the Telehealth session
   d. Other (Please specify) ______

7b. If yes, in what form do you receive the summary? (select ALL that apply)
   a. Verbal
   b. Email
   c. SMS
   d. Printed copy in post

   e. via my General Practitioner
   f. Other (Please specify) ______
8. Imagine receiving a written summary of the telehealth consultation almost immediately by the end of your video call. How useful do you think it will be?
   a. Not at all useful
   b. Not really useful
   c. Somewhat useful
   d. Very useful

Post-simulation Questionnaire for Clinicians

The seven constructs used were: performance expectancy (3 items), the degree to which one believes that using the system (CSA) will enhance job performance; effort expectancy (4 items), ease of using the application; attitude (4 items), one's overall affective reaction to using the system; facilitating conditions (3 items), the degree to which one believes that organizational and technical infrastructure supports the use of the system; self-efficacy (4 items), situation-specific belief about how well someone can execute actions for the prospective task; anxiety (4 items), one's apprehension or fear when faced with the possibility of using the system (Adapted from Computer Anxiety); and behavioural intention (3 items), one's intention or willingness to use a specific technology. We made modifications to sections of the UTAUT that were not relevant to this use case in the Australian context. The following statements were removed: 'If I use the system, I will increase my chances of getting a raise. ' (technology use is not linked to remuneration.), ii) a specific person (or group) can assist with system difficulties (support mechanisms for the product are not yet delineated), iii) all questions from the social influence domain (social influence is not relevant to the use case), iv) inserted the technology name 'CSA', where appropriate, into the questions, v) added the clarifying prefix 'assuming that the system was available as a standard feature in the telehealth platform(s) I use regularly…' to questions in the 'behavioural intention' domain, vi) specified the time horizon for questions in the 'behavioural intention' domain as 6 months.

On a scale of 'strongly disagree' to 'strongly agree', please rate the following statements to do with the possible use of the "Consultation Summary Generation Feature" in your real-world practice.
Note: When answering these questions, please consider both the consultation summary generation feature, and the medical dictionary feature.

References:

- Venkatesh, V., et al., User Acceptance of Information Technology: Toward a Unified View. MIS Quarterly, 2003. 27(3): p. 425-478.
- Davis, F.D., R.P. Bagozzi, and P.R. Warshaw, User Acceptance of Computer Technology: A Comparison of Two Theoretical Models. Management Science, 1989. 35(8): p. 982-1003.

Performance expectancy
- I would find the CSA useful in my job.
- Using the CSA would enable me to accomplish tasks more quickly.
- Using the CSA would increase my productivity.
- ~~If I use the CSA, I will increase my chances of getting a raise.~~

Effort expectancy
- My interaction with the CSA would be clear and understandable.
- It would be easy for me to become skilful at using the CSA.
- I would find the CSA easy to use.
- Learning to operate the CSA was easy for me.

Attitude toward using technology
- Using the CSA is a good idea.

- The CSA would make work more interesting.
- Working with the CSA would be fun.
- I liked working with the CSA.

Social influence

- ~~People who influence my behaviour would think that I should use the CSA.~~
- ~~People who are important to me would think that I should use the CSA.~~
- ~~The senior management of the organisation would be helpful in the use of the CSA.~~
- ~~In general, the organisation would support the use of the CSA.~~

Facilitating conditions

- I will have the resources necessary to use the CSA.
- I have the knowledge necessary to use the CSA.
- The CSA is not compatible with the other systems I use.
- ~~A specific person or group would be available for assistance with system difficulties.~~

Self-efficacy

I could generate a Patient Summary using the CSA...

- If there was no one around to tell me what to do as I go.
- If I could call someone for help if I got stuck.
- If I had a lot of time to complete the job for which the software was provided.
- If I had just the built-in help facility for assistance.

Anxiety

- I feel apprehensive about using the CSA.
- It scares me to think that I could lose a lot of information using the CSA by hitting the wrong key.
- I hesitate to use the CSA for fear of making mistakes I cannot correct.
- The CSA is somewhat intimidating to me.

Behavioural intention to use the system

Assuming that the CSA was available as a standard feature in the telehealth platform(s) I use regularly…

- I intend to use the CSA in the next 6 months.
- I predict I would use the CSA in the next 6 months.
- I plan to use the system in the next 6 months.

<u>Post-simulation Questionnaire for Simulated Patients</u>

Reflecting only on your experience today, please rank the following statements on a scale of strongly disagree to strongly agree.

Based on my experience of the simulated consultation and reading of the patient summary document that was generated, I think that:

1. The patient summary feature should be available to patients in future video-based telehealth consultations.
2. Patients will find the summary document generated from using the feature:
   a. easy to understand
   b. easy to save or print
   c. trustworthy
   d. useful (e.g. to save for their own records, share with others)
3. Patients will find the patient summary feature:
   a. usable
   b. useful
4. Patients will find the overall medical terminology explanation feature:
   a. usable
   b. useful
5. Patients will find the medical terminology explanations included in the summary document:

    a. easy to understand
    b. trustworthy
    c. useful (e.g. to understand medical terms better)
6. The patient summary generated is:
    a. an accurate and true reflection of the consultation and any agreed plans.
    b. appropriate in length and level of details.
7. The breadth and depth of information contained in the summary will be satisfactory for patients.
8. I could see myself benefiting from a summary document like this as part of my usual healthcare.

Comments: Would you like to explain any of your responses above or add further comments about the summary / your experience of the simulated consultation?

No. 4. Semi-Structured Interview Guides

For Clinicians

Commensurate with the semi-structured interview method, the questions evolved based on participants' responses during each interview conversation.

Current experience and perceptions of telehealth use and consultation summaries

1. Do you currently use video-based telehealth in your clinical practice? How often?
2. How has your experience with telehealth been (prior to today)? Do you find it a valuable means of delivering healthcare to your patients?
3. How satisfied are you with your current telehealth platform (e.g. Healthdirect Australia)?
4. Do you currently generate summaries for patients?
   a. If yes, please describe the process and any related outputs (e.g., documents, audio/video recording)?
   b. If no, do you think generating a patient summary is useful? (probe: for patient, carers, for you, for clinic,)

Experience with the consultation summary application (CSA)

5. What was your overall impression of the patient consultation summary? Do you think this feature could help your practice in the real-world? Why, or why not?
6. Could this feature better support patients' ongoing care, especially from a patient-centred perspective? Why, or why not?
7. If this feature was made available in its current form as a regular part of your telehealth software platform, would you use it in your practice? Why, or why not?
8. From a process and workflow perspective, is this feature feasible to adopt in a real-world telehealth practice?
9. Do you anticipate this feature would increase or decrease the duration of a patient consultation:
   a. on the day of the consultation? (why?)
   b. in the long run? (why?)
10. Do you anticipate this feature would increase or decrease the quality of patient care you can provide:
    a. on the day of the consultation? (why?)
    b. in the long run? (why?)
11. Do you anticipate any other efficiency gains or losses from using this feature? (e.g. less backlog of downstream note-taking / manual CSA, less context switching when preparing notes at the end of the day instead, less cognitive load to remember case details later)
12. Do you foresee any risks to patients if this feature was used in the real-world? (Consider both safety and quality of patient care)
13. Do you have any other ideas on improving the feature?

Consultation summary output

14. Do you think the patient consultation summary will be useful for patients in the real world? Why, or why not?
15. What are your impressions of the level of correctness of the summary document and its medical terminology? (Probe: spelling, quality of explanation etc.)
16. Do you have any other ideas about improving the summary document?
17. Is the summary easy to navigate, following a logical design and easily navigable structure?
18. Are the font sizes legible, typography readable, and colour contrast sufficient for text and background elements following WCAG (Web Content Accessibility Guidelines) standards?
19. Is the look and feel consistent in the navigation menu across pages if there is more than one User Interface in the application? Simplified user interface where information is presented in a clear and organised manner.

20. Is the operation of the application distracting or distressing the patient during the telehealth consult?

Experience of the research study

21. From a research process perspective, what did you think about the simulated consultation and patient personas? How could they be improved?
22. Were the role-play consultation and patient personas reflective of your real-world clinical experience? Why, or why not?
23. Do you have any other suggestions for improving our research process for next time?

<u>For Simulated Patients</u>

Commensurate with the semi-structured interview method, the questions will also evolve based on participants' responses during each interview conversation. *<u>Note</u>*: The following questions will be answered by simulated patients as themselves, with their perspectives, reflecting on the simulated consultations and patient personas (rather than in character).

Experience with the telehealth enhancement prototype

1. How was your overall experience of today's role-play teleconsultation involving the patient consultation summary feature?
2. What did you like/dislike about the telehealth experience involving this consultation summary feature?
3. How would you compare your experience today with other telehealth experiences you may have had in the past? (if applicable)
4. Do you think this application could work in a real-world consultation? Why, or why not?
5. Do you foresee any risks to patients if this application was used in the real-world? (Consider both safety and quality of patient care)

Consultation summary output

6. Do you think the patient summary will be helpful for patients in the real world? Why, or why not?
7. Overall, do you think a real patient would be satisfied with such a summary? (Probes: readability, quality of the medical terminology explanation, usability of the medical terminology hovering feature, information contained in the URL link)
8. Are there any parts of this summary that you did not understand or would want to be clarified (and why)?
9. How useful was it that you found the medical terminology explanation feature on screen and in the summary appendix?
10. Do you have any other suggestions for improving the summary document?
    (Consider layout/format, length, language, and content issues not addressed above.)

Experience of the research study

11. From a research process perspective, how did you find the simulated consultation and patient persona? What did you like/dislike about it?
12. How was your experience acting as a patient? Could we have given you any other information to help you better perform as a pretend patient? (e.g. more details in the persona)
13. Do you have any other suggestions on improving our research process for next time?

No. 5. Supplementary Table 1: Improvements Implemented to the CSA

This table highlights areas identified about the CSA through the clinical simulation, clinicians' comments or feedback, and how the research team addressed them by tweaking the CSA's features.

Supplementary Table 1. Overview of how participant feedback was incorporated into the CSA

Areas	Identified within the simulation	Clarified post-simulation from the interview	Considerations addressed by technology research and development team
**Mode of input (typing or voice dictation)**	Pre-simulation, all participants both typing and voice dictation.<br><br>During the simulated consultation, one clinician did not input anything (C4). The others all typed and one of them also used the voice dictation (C5).	All found the voice dictation mode is cumbersome compared to typing as it is what most clinicians are used to in practice. They also found the transcription version lacked accuracy, requiring them to correct typos especially regarding names and medication details. (C1, C2, C5, C6, C7).<br><br>Those familiar with transcription found the CSA's lack of support for voice commands (e.g., new line, comma, etc) difficult to use. (C1, C7).<br><br>The adoption of automatic transcription and generation of summary document with the help of AI might be helpful. (C2, C6, C7).	The voice dictation feature was made optional through a toggle on/off in the settings.
	What clinicians expected to be presented as bullet points (e.g., by putting a dash before a point and written as a fresh line for these points) were reflected as one bulk paragraph in the document summary (C1, C7)	Request for a rich text format input capability. (C1, C6)<br><br>Drawing tables would also be beneficial, especially when writing medications and their descriptions (e.g., indication, dose, frequency, etc.) (C7).	Rich-text formatting capability was introduced (bold, italic, underline, number and dot bullet points). Ability to add tables was added later on.

Areas	Identified within the simulation	Clarified post-simulation from the interview	Considerations addressed by technology research and development team
**Patient access to summary**	One clinician shared the summary preview with the patient but did not explain how to access the summary, and patients left the consultation without it. (C2)	Relying only on the summary saved as a PDF or printed during the call is a potential risk (C5, C7). In cases where a call is terminated prematurely (e.g., connection disruption, technical difficulties requiring a redialling, or wrong press of the end call button), there is no way to access the summary written so far. This will pose expectations for clinicians to recreate the summary. If this occurs often enough, it will frustrate the clinicians (C6, C7).<br><br>Emailing the patients the PDF gives clinicians control and email is universally used (C5). Further, it would be helpful to send the summary into the EMR via email in one click or a 'copy' and 'paste' option (C3, C6).	Emailing the summary feature developed earlier was re-enabled.
**User interface and user experience of the application**	When initiated, the CSA came to the foreground to replace the patient's video frame. This happened for the duration of typing and sharing the summary view, which for all cases were throughout the whole duration of the consultation.	It is currently not possible to drag and drop the headings or text fields to reorder them (C1).<br><br>Clinicians could not adjust the video size, which some found problematic as they preferred a larger screen to observe the patient closely (C5). Therefore, while the large size of the application might invite engagement from the clinician, it redirected their focus to the application.<br><br>C3 mentioned that throughout medical training, it is considered best practice to maximize eye-contact	The app was redesigned in the form of a "drawer app" which floats over the video with ability to toggle on/off to enable dragging and resizing of the CSA window.

Areas	Identified within the simulation	Clarified post-simulation from the interview	Considerations addressed by technology research and development team
		with the patients and minimize typing and facing away from the patients.	
	The definition of words in the medical dictionary can be lengthy and takes up space in the summary's on-screen estate which might reduce salience of the clinician's notes. For example, in a two and a half pages summary document, one and a half page is for the definitions alone for 'lung cancer', 'chemotherapy', 'radiotherapy', 'prognosis', 'pain', 'constipation', 'fatigue'(P1). It also does not allow clinicians to edit the definitions.	Clinicians would not have the time to proofread all the terminology definitions. There could be something that potentially conflicts with the clinician's message, adds confusion, or directs the patient to do something differently, which would be unhelpful.	A limit character was introduced to the definition.

No. 6. Run Sheet

<u>For Clinicians</u>

Introductions

Hello, Dr. "XYZ." Welcome. Thank you for making time to meet with us today.
My name is "XYZ", and I am a researcher at the University of Melbourne. I will be looking after you today. I also want to introduce you to my colleague "XYZ", who is a technical expert. "XYZ" will be in a breakout room with us in case of any technical issues.

Clinician Breakout Room

Session overview
I understand you have read the plain-language statement and signed the consent form. I want to reiterate that your participation today is voluntary. If at any point, you wish to stop the session, please let me know. We can also take breaks if you need to. Before we start, can I please confirm that you are happy to participate in the session today and have it recorded? We will de-identify all the data, including your personal information, before publishing the results. Only approved researchers will have access to your personal information.

Now it is recording. Let's run through briefly what you can expect from this session. Today, we would like to test your experience on a telehealth feature, which I will refer to as the tool in this study. We anticipate that this session will take no longer than 90 minutes.

We will show you a short video on what the tool is and how to interact with it. Then, we will give you the login details to play around with the tool. Once you are comfortable with the tool, we will show you the patient referral, which is a persona that will be acted by a professional simulated patient, and you will conduct a simulated consultation based on that persona on the telehealth platform. After that, we will conduct a debriefing interview about your experience. Does that all sound good?

I will take you through all the steps one by one, so please don't worry. I will be here with you to guide you through the whole process. One thing I'd like to note is that this is a new tool we're testing. So, some technical glitches are to be expected. Thank you.

The first thing we want to do is understand how you use telehealth in your current medical practice. For that, we have developed a survey. I am pasting the survey link in the chat. Please click the link and complete the survey using your best estimate. It shouldn't take more than 5 minutes. I will be here if you have any questions. Feel free to turn off your camera and audio if you prefer.

The next step is to introduce you to the tool we are trying to test. We have a short instructional video that you can watch first. Then we will also let you play with the tool yourself. In this way, we hope you will start to feel somewhat comfortable with the tool before we go into the simulated consultation with the patient actor. So, I will paste a link to the video on the chat for you. Please click on the link and watch the video. It takes about three minutes. You are very welcome to pause the video / rewind it, etc., if you need to, and again, feel free to turn off your camera and mute yourself if you prefer.

Now we will have a play with the tool. For that, we would like you to share your screen. Before you do that, please make sure any sensitive or private information you don't want us to see is closed. If you need to remove any items from your space, you are welcome to do so. Please take your time. *(If the participant struggle to share the screen) → Are you able to share your full desktop screen? I can walk you through the steps. On the bottom of your Zoom screen, do you see the screen share button at the bottom of the Zoom> select "desktop" > at the bottom left, and check the box "share*

sound". Thank you. Now, we can help you play with the tool. For that, I am pasting a link to the tool and password for you.

Once the clinician is on the platform, instruct them accordingly.

- Please look at the grey menu on the far left, you will see a button called 'enter' next to your name. Please click on that.
- Go to the green Apps and Tools button at the bottom right > Record patient summary.
- As you can see in the video, this is where you generate the consultation summary. Please have a play with it.
- For the purpose of this study, it doesn't matter which title you choose.
- Now imagine if you were to use this tool as part of a consultation, how might you use it? As you can see in this form, you can both type directly in the box or record using the red buttons on the right, and the tool will auto-transcribe. Feel free to use the tool as it comes naturally to you.
- However, we do suggest playing with both the typing and recording functions if you can. You might find one or the other easier or more difficult, which is fine. That's exactly what we want to understand: which option you liked better.
- Please enter some mock information in the form either by typing or recording, so you can get a sense of how it plays out. Use the word pain at least once in the background, as a medical term, as a function for patients to hover over it to view the definition in the summary.
- When you are ready to share the summary with the patient, you could advise them to hover over some medical terminology for more information. Please note that you can change the definition if you like. The patient will find the terminology at the bottom of the page if they save or print a PDF.
- You can also invite the patient to save the summary on their end, since once you close the page without saving, they won't have access to it anymore.

Before we begin the consultation, I'd like to ask briefly about your workflow when conducting telehealth.

- Do you use an EMR system for your telehealth consultations in your practice?
- Is that where you take notes?
- Do you have a second screen?

We have created a mock EMR system for this purpose. You are welcome to use it just as if you were in your normal practice. Although, of course, because it's a mock system, you are not going to be able to look up patients' data except for the referral letter. I have pasted the link to the EMR system here in the chat.

Now we will have a simulated consultation in which you will use the tool during a mock consultation. Are you feeling somewhat comfortable using the tool during a mock consultation now? I want to reiterate that this mock EMR is simply to mimic your clinical workflow. We are not really testing the EMR as part of today's study. So, please engage with it as needed. However, please note that today's study focuses on the transcription tool. And we are mainly interested in your perceptions of that tool. The simulated consultation will be with a simulated patient. Now, let's have a look at the referral letter to help you prepare for the mock consultation. I will paste a link in the chat for you– please have a read of the referral letter.

We will now move into the simulated consultation with the simulated patient. Imagine you were having a consultation with this patient and using the consultation summary tool as part of it. Please use the tool as if it were part of your usual medical practice. We would love to hear your views on the tool afterwards. I will be here the whole way through if you have any questions.

Are you happy for us to proceed with the simulated consultation? If you like, we can take a short break now. Great, now I will talk you through the steps to start the patient consultation. First, can you please close this window?

Now, if you go back to the HDA home page where we first landed, you can see that the patient is already online. Before we enter the patient room, I would like you to turn off your Zoom video, as the patient will see you via the HDA video instead. Great, thanks! When you enter the patient room in a minute, I will hang around for a few minutes to ensure you and the patient actor can hear and see each other. Once we know that the tech is working, I will switch off my video and audio. I suggest as soon as the consultation starts, you have the consultation summary tool open. However, how you use it in the consultation is totally up to you, and what comes naturally to you as part of your clinical workflow. When you are done with the telehealth consultation, please close the HDA tool. But please do stay back on Zoom. At the end, we will have a debrief about your experience. Does that all sound ok? Great, now can you please click on the green button next to the patient's name?

Quick test of audio and video on HDA

Please ask the patient if they can see you and hear you? And can you see and hear the patient?
<If any issues, help troubleshoot> Great, I will keep my audio and video off while you talk to the patient to avoid distractions. But, please do yell out if you have any questions or need any help. All the best!

After you finish the consultation, please stay on the call. The patient will drop off. However, it would be great if you and I could do a debrief. We will request that you complete a short post-survey and a debriefing interview.

<u>For Simulated Patients</u>

Introductions

Hello, "XYZ." Welcome. Thank you for making time to meet with us today.
My name is "XYZ", and I am a researcher at the University of Melbourne. I will be looking after you today. I also want to introduce you to my colleague "XYZ", who is a technical expert. "XYZ" will be in a breakout room with us in case of any technical issues.

Would you like to introduce yourself briefly? Your name, a brief sharing of your experience as a simulated patient (e.g., how long, for whom, the kind of cases). Thank you. Let's start with the briefing. With that, I will take you to a breakout room where we can help you get set up. Bear with me one minute while I set us up.
-- Go into the breakout room.

Session overview
I understand you have read the plain-language statement and signed the consent form. I want to reiterate that your participation today is voluntary. If at any point, you wish to stop the session, please let me know. We can also take breaks if you need to. Before we start, can I please confirm that you are happy to participate in the session today and have it recorded? We will de-identify all the data, including your personal information, before publishing the results. Only approved researchers will have access to your personal information.

Now it is recording. Let's run through briefly what you can expect from this session. Today, we would like to test your experience on a telehealth feature, which I will refer to as the tool in this study. We anticipate that this session will take no longer than 90 minutes. Today's session will involve a series of activities, including two surveys, a simulated consultation, and a debrief interview. I will take you through all the steps one by one. So, please don't worry. I will be here with you to guide you through the whole process. I want to reiterate that this is a new tool we are testing.

The first thing we want to do is understand how you use telehealth. For that, we have developed a survey. Please note that at this point, you are to fill in the survey as yourself, not based on any

personas. I am pasting the survey link in the chat. Could you please complete this survey now? Please answer the questions based on your best estimate. It will take less than 5 mins. Let me know when you are done. Feel free to turn off your video and audio as you fill in the survey. I will be here if you have any questions.

Soon, we will have a simulated consultation from this room, conducted on this computer, in which you will act as a patient with the persona you've been given. The consultation will be with a real palliative care / medical oncologist clinician. The consultation may take between 15 to 40 minutes, depending on how it goes. Can I confirm whether you have received the persona on which today's simulated consultation will be based? Here is a printed version of the persona. Feel free to read it again if you like, and ask me any questions you have. Do you feel comfortable acting in character today? The clinician will not have read the persona, but will get a referral letter about your general condition. The clinician will likely ask you questions about your medical journey (diagnosis, treatments you have received so far, symptoms you are experiencing, and what you hope to achieve). Please feel free to improvise as you need to as you might not know the answers when put in the spot. If you are not sure about anything, feel free to post a question in the Zoom chat, and we will respond. We trust your judgment. We recommend that you have your persona handy for the simulated consultation, it is printed here. Please note that the clinician will be testing a telehealth tool today. This tool is in progress, so if the clinician gets flustered, that is to be expected and is a finding in itself. We really appreciate your patience and understanding if and when that happens.

We will now move into the simulated consultation with the clinician. Let's first have you click this <link> to enter the telehealth waiting room. Please enter your name on the persona.

Before you join the consultation room with the clinician, I would like you to switch off your Zoom video, as the clinician will see you via the telehealth tool instead. Great, thanks! When you enter the consultation room in a minute, I will hang around for a few minutes to ensure you and the clinician can hear and see each other. Once we know that the tech is working, I will leave this room and stay next door, switch off my video and audio.

When you are done with the telehealth consultation, please close the telehealth tool. Please follow the clinician's instructions after the telehealth. But please do stay back on Zoom. At the end, we will have a debrief about your experience. Does that all sound ok?

Quick test of audio and video on HAD. Please ask the clinician if they can see you and hear you? And can you see and hear the clinician? <If any issues, help troubleshoot>

Great, I will keep my audio and video off while you are talking to the clinician, to avoid any distractions. But please do yell out if you have any questions or need any help. All the best!
